# Dose‐dependent increases in p70S6K phosphorylation and intramuscular branched‐chain amino acids in older men following resistance exercise and protein intake

**DOI:** 10.14814/phy2.12112

**Published:** 2014-08-08

**Authors:** Randall F. D'Souza, James F. Markworth, Vandre C. Figueiredo, Paul A. Della Gatta, Aaron C. Petersen, Cameron J. Mitchell, David Cameron‐Smith

**Affiliations:** 1 Liggins Institute, University of Auckland, Auckland, New Zealand; 2 Centre for Physical Activity and Nutrition, School of Exercise and Nutrition Sciences, Deakin University, Victoria, Australia; 3 Institute of Sport, Exercise and Active Living (ISEAL), Victoria University, Melbourne, Victoria, Australia

**Keywords:** Leucine, mammalian target of rapamycin, mass spectrometry, muscle mass, protein synthesis, sarcopenia

## Abstract

Resistance exercise and whey protein supplementation are effective strategies to activate muscle cell anabolic signaling and ultimately promote increases in muscle mass and strength. In the current study, 46 healthy older men aged 60–75 (69.0 ± 0.55 years, 85.9 ± 1.8 kg, 176.8 ± 1.0 cm) performed a single bout of unaccustomed lower body resistance exercise immediately followed by ingestion of a noncaloric placebo beverage or supplement containing 10, 20, 30, or 40 g of whey protein concentrate (WPC). Intramuscular amino acid levels in muscle biopsy samples were measured by Gas Chromatography–Mass Spectrometry (GC‐MS) at baseline (before exercise and WPC supplementation) plus at 2 h and 4 h post exercise. Additionally, the extent of p70S6K phosphorylation at Thr389 in muscle biopsy homogenates was assessed by western blot. Resistance exercise alone reduced intramuscular branch chain amino acid (BCAA; leucine, isoleucine, and valine) content. Supplementation with increasing doses of whey protein prevented this fall in muscle BCAAs during postexercise recovery and larger doses (30 g and 40 g) significantly augmented postexercise muscle BCAA content above that observed following placebo ingestion. Additionally, the fold change in the phosphorylation of p70S6K (Thr389) at 2 h post exercise was correlated with the dose of whey protein consumed (*r* = 0.51, *P* < 001) and was found to be significantly correlated with intramuscular leucine content (*r* = 0.32, *P* = 0.026). Intramuscular BCAAs, and leucine in particular, appear to be important regulators of anabolic signaling in aged human muscle during postexercise recovery via reversal of exercise‐induced declines in intramuscular BCAAs.

## Abstract (precis)

In older men, p70S6K phosphorylation is increased in a dose‐dependent manner with increasing doses of whey protein intake after resistance exercise. Resistance exercise reduces intramuscular concentrations of branched‐chain amino acids but whey protein intake post exercise maintains these concentrations.

## Introduction

Skeletal muscle mass is tightly regulated by the balance between rates of muscle protein synthesis (MPS) and muscle protein breakdown (MPB). Increases in MPS induced by both resistance exercise and protein feeding are thought to be mediated primarily though the mammalian target of rapamycin (mTOR) pathway. p70S6 kinase (p70S6K) is a major downstream component of the mTOR pathway which is responsible for the initiation of protein translation and is thus often used as a proxy measure for MPS (Drummond et al. 2009). Amino acids (AAs), and leucine in particular, have been proposed to be the primary nutritional regulators of the mTOR signaling pathway cumulating in increases in MPS (Smith et al. 1992; Atherton et al. 2010). MPS appears to be much more tightly regulated when compared to MPB, and for this reason, MPS and associated anabolic signaling often serve as the primary measures in relation to the influence of protein feeding and resistance exercise on muscle protein turnover (Glynn et al. 2010). In many cases, acute p70S6K phosphorylation correlates with long‐term changes in muscle size (Baar and Esser 1999; Terzis et al. 2008; Mayhew et al. 2009; Mitchell et al. 2013). However, there are also reports of a lack of correlation between acute MPS or p70S6K and hypertrophy responses within the same subject, suggesting that additional mechanisms may be involved (Mitchell et al. 2012, 2014). Nevertheless, multiple studies which have tested the acute (MPS and p70S6K phosphorylation) and chronic (hypertrophic) effects of the same exercise or nutritional manipulation in different subjects do tend to show very similar patterns of group changes (Hartman et al. 2007; Wilkinson et al. 2007; West et al. 2009a,b; Burd et al. 2010a,b; Mitchell et al. 2012, 2013).

The ingestion of graded amounts of high‐quality protein such as whey after resistance exercise has been shown to increase MPS in a dose‐dependent manner. In young men (20–30 years), there appears to be a ceiling above which no further augmentation of MPS occurs in response to protein ingestion. For example, a plateau in MPS occurs following ingestion of 20 g of either egg (Moore et al. 2009) or whey (Witard et al. 2014) protein in young male subjects, with no greater MPS response observed in response to surplus ingestion of 40 g protein. However, multiple studies have shown that older adults (>60 years) exhibit a lower anabolic signaling and MPS response to protein feeding, resistance exercise, and the combination of feeding and exercise when compared to young men (Cuthbertson et al. 2005; Fry et al. 2011; Burd et al. 2013). This phenomenon termed “anabolic resistance” is exemplified by a recent study by Yang et al. (2012b) which showed that MPS in older men following resistance exercise increased to a greater extent after the ingestion of 40 g of whey protein when compared to the response elicited by 20 g whey protein (Yang et al. 2012b). Nevertheless, the magnitude of the MPS response to feeding 40 g of protein was slightly less in older men than that which was observed in young men who were fed a maximally effective 20 g dose of protein (Yang et al. 2012a; Churchward‐Venne et al. 2013b). It appears that deficits in feeding‐induced p70S6K phosphorylation may at least partially underpin anabolic resistance in aged skeletal muscle (Cuthbertson et al. 2005). Much less is known about the effect of graded whey protein ingestion on p70S6K phosphorylation in older human subjects, which is the putative mechanism underlying nutrient‐induced increase in MPS.

It is well established that ingestion of amino acids (AA) or intact protein leads to dose‐dependent increases in circulating plasma essential amino acids (EAAs; Bohe et al. 2003; Moore et al. 2009; Yang et al. 2012b; Witard et al. 2014). On the other hand, much less is known about the effect of resistance exercise and protein feeding on the concentrations of AAs within the muscle tissue itself. A classic study by Bohé et al. showed a stronger relationship between plasma EAAs and MPS when compared to intramuscular EAA concentrations (Bohe et al. 2003). However, the concentrations of the individual EAAs were not reported independently in this study (Bohe et al. 2003). The authors hypothesized that MPS was primarily regulated by extracellular rather than intracellular EAA availability. However, it has since been demonstrated that among the EAAs, leucine one of the three Branched‐Chain Amino Acids (BCAAs), appears to be the primary trigger of amino acid‐induced anabolic signaling and MPS (Atherton et al. 2010). Moreover, recent mechanistic work suggests that the activation of mTOR in response to leucine is sensed through intracellular proteins such as the leucyl‐tRNA synthetase (Han et al. 2012) and/or the ragulator (Zoncu et al. 2011). Consistently, recent work has also suggested that the pattern of change in BCAAs in response to resistance exercise and protein feeding may be different when compared with the other EAAs (Drummond et al. 2010, 2011; Churchward‐Venne et al. 2012, 2014).

The purpose of the present study was to characterize the changes in intramuscular levels of EAAs and BCAAs in response to resistance exercise and graded ingestion of whey protein in older men. A secondary aim was to determine the effects of graded intakes of whey protein on the extent of exercise‐induced phosphorylation of p70S6K at Thr389 as a marker of mTOR pathway activation in the muscle of older men and to examine the relationship between intramuscular EAA/branch chain amino acid (BCAA) levels and growth signal transduction via the mTOR pathway.

## Methods

### Subjects and ethical approval

Forty six healthy elderly untrained men (60–75 years of age) volunteered to participate in this study, subject characteristics are reported in Table 1. Individuals participating in any regular resistance exercise training program (two or more days per week) and/or with preexisting metabolic or cardiovascular diseases were excluded. Individuals who engaged in aerobic exercise up to three times per week and/or were taking anticoagulation or antihypertensive medication were not excluded from participation, however, those on aspirin/fish oil supplements were requested to abstain from these medications throughout the experimental trial. Prior to any experimental procedures, subjects were provided with oral and written information regarding the experimental protocols and potential risks involved and written consent to participate was obtained. Randomization to place immediately after consent was obtained. All experimental procedures employed by this study were carried out in coherence with the Helsinki declaration and were formally approved by the Deakin University Human Research Ethics Committee.

**Table 1.** Subject characteristics. Means ± SD

	Placebo	10 g Whey	20 g Whey	30 g Whey	40 g Whey
*n*	15	7	7	7	10
Age (years)	67.8 ± 3.9	71.1 ± 4.9	69.3 ± 3.6	70.0 ± 3.3	68.1 ± 4.1
Body mass (kg)	89.6 ± 14.0	82.8 ± 8.3	90.5 ± 19.5	79.6 ± 9.0	84.7 ± 11.8
Height (m)	1.80 ± 0.04	1.74 ± 0.04	1.78 ± 0.10	1.72 ± 0.06	1.78 ± 0.10
BMI (kg/m^2^)	27.5 ± 4.6	27.4 ± 3.3	28.6 ± 5.4	27.4 ± 3.0	26.5 ± 3.3
1‐RM Squat (kg)	84.1 ± 33.0	59.6 ± 11.3	81.0 ± 26.9	67.9 ± 19.6	90.8 ± 39.9
1‐RM Leg press (kg)	177.7 ± 73.5	175.9 ± 28.2	211.9 ± 111.6	183.4 ± 32.9	205.4 ± 66.4
1‐RM Leg Ext. (kg)	53.01 ± 21.9	52.6 ± 10.6	49.9 ± 29.9	56.0 ± 10.2	68.7 ± 21.1

### Study design

Approximately 1 week prior to the experimental trial day, participants performed a familiarization session during which one‐repetition maximum (1RM) strength testing was also performed to determine the experimental exercise load (80% of 1RM). The maximal weight that subjects could lift for 3–6 repetitions (3–6 RM) on the bilateral barbell squat, leg press, and leg extension exercises was determined and participants 1RM was estimated using the Brzycki equation (1RM = 100 × load rep/(102.78–2.78 × reps completed; Reynolds et al. 2006). Participants were instructed to abstain from any vigorous physical activity in the week between the familiarization and the experimental trial day. The evening before the trial, participants ingested a standard evening meal (2103 kJ, 54% carbohydrate, 29% fat, 17% protein) before 10 pm and were instructed to eat nothing afterward. The following morning, the subjects arrived at (~7 am) in a fasted state. Participants were randomly allocated into one of five treatment groups; noncaloric placebo (*n* = 15), 10 g whey (*n* = 7), 20 g whey (*n* = 7), 30 g whey (*n* = 7), and 40 g whey (*n* = 10).

### Resistance exercise and supplementation trial

Upon arrival at the laboratory, individuals rested in a supine position for ~30 min prior to collection of resting muscle biopsy samples (see below section). Participants then rested supine following collection of resting muscle biopsy for approximately 10–15 min after which time the exercise protocol commenced. The exercise protocol began with a 10‐min warm‐up involving light cycling on a bicycle ergometer and a single low load warm‐up set for each of the three resistance exercises. Participants then completed three sets of 8–10 repetitions of bilateral barbell smith rack squat, 45° leg press, and seated knee extensions at 80% of their predetermined 1RM. The exercises were performed in a circuit manner with 1 min rest between each exercise and 3 min rest between subsequent sets, the exercise protocol took approximately 20 min to complete. Following completion of the exercise protocol, subjects were immediately provided with a fixed‐volume (350 mL) beverage, containing a flavored noncaloric placebo, or one of the four doses of whey protein concentrate (10 g, 20 g, 30 g, or 40 g). Subjects were instructed to ingest the beverage within 2 min and were required to ingest the total volume provided. Following consumption of the supplements, subjects rested in a supine position throughout the 4 h of postexercise recovery with additional muscle biopsy samples collected at 2 and 4 h post exercise.

### Muscle biopsies

Muscle biopsies (~100 mg) were collected from the vastus lateralis muscle under local anesthesia (1% Xylocaine) using a Bergstrom needle modification of manual suction. All three biopsies were collected from the same limb starting distally and moving proximally. A gap of at least 2–3 cm between sequential biopsies was maintained in order to avoid any potential confounding effects caused by repeated sampling for the same location. Biopsies were quickly frozen in liquid nitrogen and stored at −80°C until further analyses.

### Western blotting

Muscle tissue (~50 mg) was homogenized in ice‐cold RIPA lysis buffer (50 mmol/L Tris‐HCl, pH 7.4, 150 mmol/L NaCl, 0.25% deoxycholic acid, 1% NP‐40, 1 mmol/L EDTA supplemented with a cocktail of protease and phosphatase inhibitors including 1 mmol/L PMSF, 1 *μ*g/mL aprotinin, 1 *μ*g/mL leupeptin, 1 mmol/L Na_3_VO_4_, and 1 mmol/L NaF). The resulting homogenates were agitated for 1 h at 4°C, centrifuged for 15 min at 13,000 *g*, and the protein content of the supernatant was determined using a BCA‐protein assay kit (Pierce, Rockford, IL) following the manufacturer's instructions. Aliquots of protein homogenate containing 50 *μ*g of total protein were prepared, mixed with Laemmli buffer, boiled, and subjected to SDS/PAGE. Proteins were separated on an 8% gel and wet‐transferred to a polyvinyl difluoride (PVDF) membrane. Following transfer, membranes were blocked in 5% bovine serum albumin (BSA)/Tris Buffer Saline/0.1% Tween 20 (TBST) for 1 h, followed by overnight incubation at 4°C with primary antibody against p‐p70S6K (Thr389; 1:1000, Cell Signalling, Danvers, MA). The following morning, membranes were washed for 30 min with TBST and probed with a goat anti‐rabbit IgG conjugated to horseradish peroxidase (HRP) secondary antibody (1:2000) for 1 h at room temperature. Membranes were then washed for 30 min in TBST and protein bands were visualized using Western Lighting‐enhanced chemiluminescence reagent (PerkinElmer Life Sciences, Boston, MA). Signals were captured using a Kodak Digital Science Image Station 440CF (Eastman Kodak Company, Rochester, NY), and densitometry band analysis undertaken with Kodak Molecular Imaging Software (Version 4.0.5; Eastman Kodak Company). Phospho‐p70S6K (Thr389) probed membranes were stripped and reprobed for Total p70S6K (1:1000, Cell Signalling) using Restore Western Blot Stripping Buffer (Pierce). Due to large changes observed in the electrophoretic mobility of the Total p70S6K protein in highly phosphorylated postexercise and supplementation muscle samples it was difficult to accurately quantify total p70S6K. This large magnitude mobility shift in total p70S6K in samples with large degrees of p70S6K phosphorylation has been previously described (Karlsson et al. 2004). Total ERK2 (ERK1/2 Cell Signalling, 1:1000) was thus used as a loading control because it did not change in any condition. Loading normalized western blot data was calculated as fold change against subjects respective baseline sample which was always ran in contiguous lanes on the same gel.

### Metabolomics

Muscle tissue (~50 mg) was homogenized in 500 *μ*L of ice‐cold 50% methanol spiked with 20 *μ*L of 10 mmol/L D‐4 Alanine (as an internal standard) using 2.8 mm ceramic beads and a bead mill homogenizer (OMNI RUPTOR 24, 5.65 m/sec, 30 sec). The muscle homogenate was transferred to a new tube, vortexed vigorously for 1 min, and frozen rapidly on dry ice. This process was repeated through two more freeze–thaw cycles. Samples were then centrifuged at 3000 *g* for 10 min, the supernatant was collected in a 15 mL falcon tube and placed on dry ice. The remaining tissue pellets were resuspended in 500 *μ*L of 80% methanol and vigorously vortexed for a further 1 min. The suspension was centrifuged at 8000 *g* for 10 min and the resulting supernatant was pooled with that from the first centrifugation and the remaining pellet was discarded. The volume of the pooled supernatant was increased to ~5–6 mL using milli‐Q water, thoroughly mixed, and were frozen at −80°C. Frozen samples were evaporated to dryness overnight using an SC250 Express SpeedVac Concentrator and RVT4104 Refrigerated Vapor Trap (Thermo Scientific Savant, San Jose, CA). Dried sample extracts were stored at −80°C until subsequent derivatization in preparation for GC‐MS analysis.

### Methyl chloroformate (MCF) derivatization

Methyl chloroformate derivatization was based on the previously optimized protocol (Smart et al. 2010). Freeze‐dried samples were resuspended in 200 *μ*L of 1 mol/L NaOH. Resuspended samples were transferred to silanized reaction tubes to which 167 *μ*L of methanol and 34 *μ*L of pyridine and 20 *μ*L of MCF were then added. Samples were vortexed for exactly 30 sec, and this process was repeated once. To separate the MCF derivatives from the reactive mixture, 400 *μ*L of chloroform was added to the mixture and vortexted for 10 sec. Thereafter, 400 *μ*L of 50 mmol/L NaHCO3 was added and vortexed for 10 sec. The upper aqueous phase was removed with a glass Pasteur pipette and 100 mg of anhydrous Na2SO4 was added to dry the organic solution. Once dried, the remaining chloroform solution was transferred to a Gas Chromatography–Mass Spectrometry (GC‐MS) vial.

### GC‐MS analysis and metabolites identification

Gas Chromatography–Mass Spectrometry (GC‐MS) instrument parameters were based on those reported by [Smart et al. (2010)]. The instrument used was an Agilent 7890A gas chromatograph coupled to an MSD‐5975C inert mass spectrometer with a split/splitless inlet. Using a CTC PAL autosampler, 1 *μ*L of sample was injected into a glass split/splitless 4 mm ID straight inlet liner packed with deactivated glass wool (Supelco). The inlet was set to 290°C, pulsed splitless at 180 kPa for 1 min, pressure of 56.76 kPa, and column flow of 1.0 mL/min, which gave a calculated average initial linear velocity of 35 cm/sec. 1.1 min after injection, the purge flow was set to 25 mL/min. The capillary column used was ZB1701 (Zebron, Phenomenex, North Shore City, New Zealand) with 30 m × 250 *μ*m × 0.15 *μ*m film thickness. (86% dimethylpolysiloxane, 14% cyanopropylphenyl, Phenomenex). Carrier gas was ultra‐high‐purity grade helium (99.9999%, BOC). GC oven temperature programing started isothermally at 45°C for 2 min, increased 9°C/min to 180°C, held 5 min; increased 40°C/min to 220°C, held 5 min; increased 40°C/min to 240°C, held 11.5 min; increased 40°C/min to 280°C, and held 2 min. The transfer line to the MSD was maintained at 250°C, the source at 230°C and quadropole at 150°C. 5.5 min into the run, the detector was turned on and ran in positive‐ion, electron‐impact ionization mode, at 70 eV electron energy, with electron multiplier set with no additional voltage relative to the autotune value. To monitor instrument carryover, N‐hexane blanks were run for every 10–12 samples. Identification of compounds was carried out using mass spectra acquired in scan mode from 38 to 550 amu, with detection threshold of 100 ion counts (Smart et al. 2010). The raw values from the GC‐MS were corrected to the spiked internal standard (D‐4 Alanine) and the wet mass of the tissue samples. Values are expressed as a fold change from the rested and fasted values because internal standards of each amino acid were not used and it was not possible to calculate absolute concentrations of AAs.

### Data analysis

Data are presented as mean ± standard error of the mean (SEM). Outliers were removed using automated stringent outlier testing (ROUTs 0.1%) in GraphPad Prism6 software (GraphPad Software, Inc., La Jolla, CA) (Motulsky and Brown 2006). Cleaned data were analyzed using a two‐way analyses of variance (ANOVA; treatment × time) with repeated measures over time. Following significant main or interaction effects, Holm–Sidak post hoc tests were used for pairwise comparison between time points and supplement groups. In addition to quantitative statistical analysis, a heat map of the data showing percentage change in various amino acids from fasting levels was generated using the R program gplots package (heatmap.2 function) to identify patterns of relative changes in amino acids over time in each supplementation group. Pearson product moment coefficients were calculated to assess the relationships between variables; a linear regression was used to determine the relationship between whey protein dose and P70S6k phosphorylation. ANOVAs and correlations were calculated using Sigmaplot 12.5 (Systat Software Inc., San Jose, CA) Statistical significance was set at *P* < 0.05 level.

## Results

### Intramuscular amino acids

**Figure 1.**
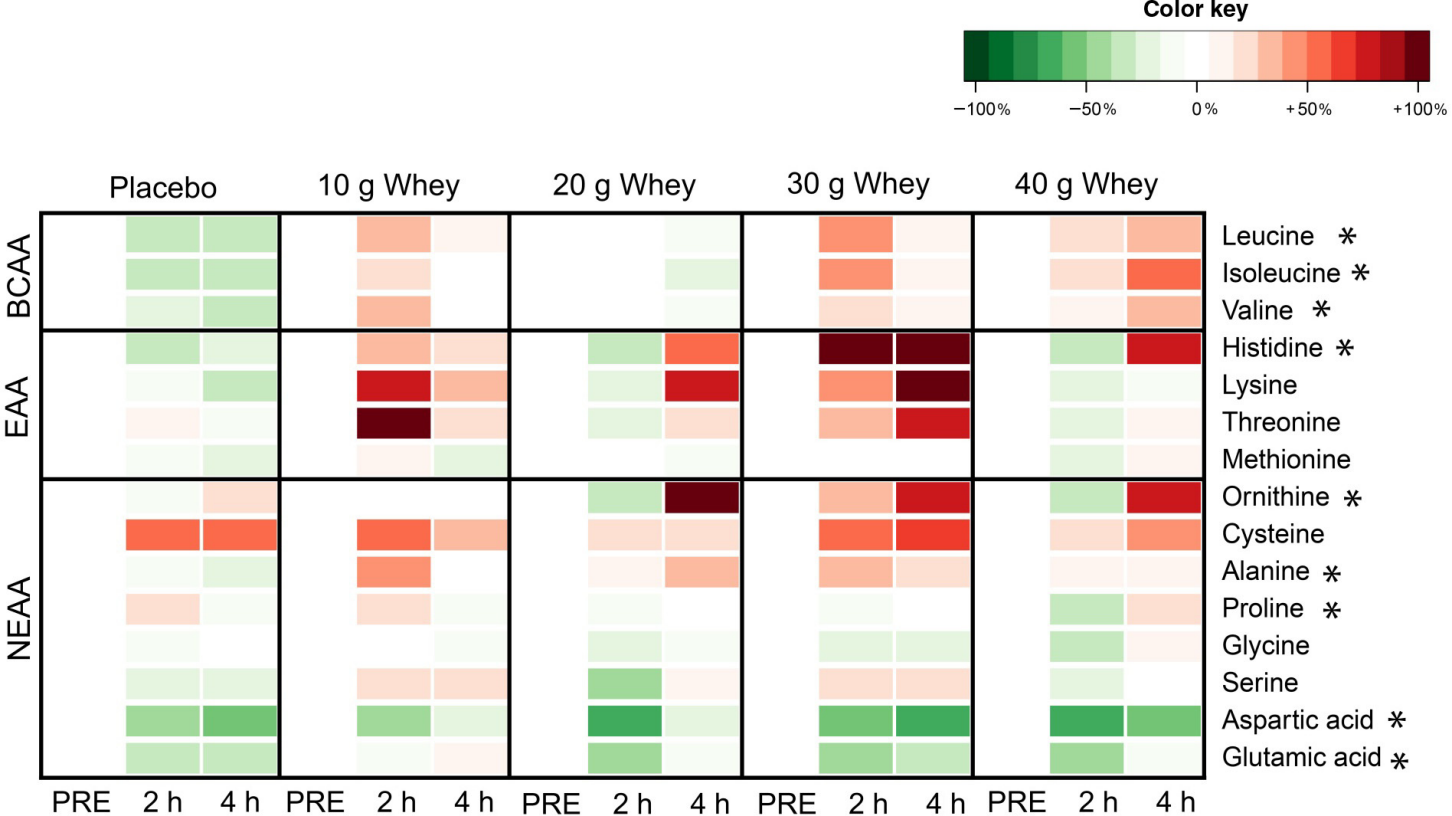
Intramuscular amino acids. This figure is a heat map which shows groups means fold changes from the resting fasted condition. Green represents a decrease in amino acid content, white represents no change, and red represents an increase in amino acid content. *significant effect revealed by ANOVA *P* < 0.05. Specific pairwise differences are omitted for clarity but are reported in the results section.

**Figure 2.**
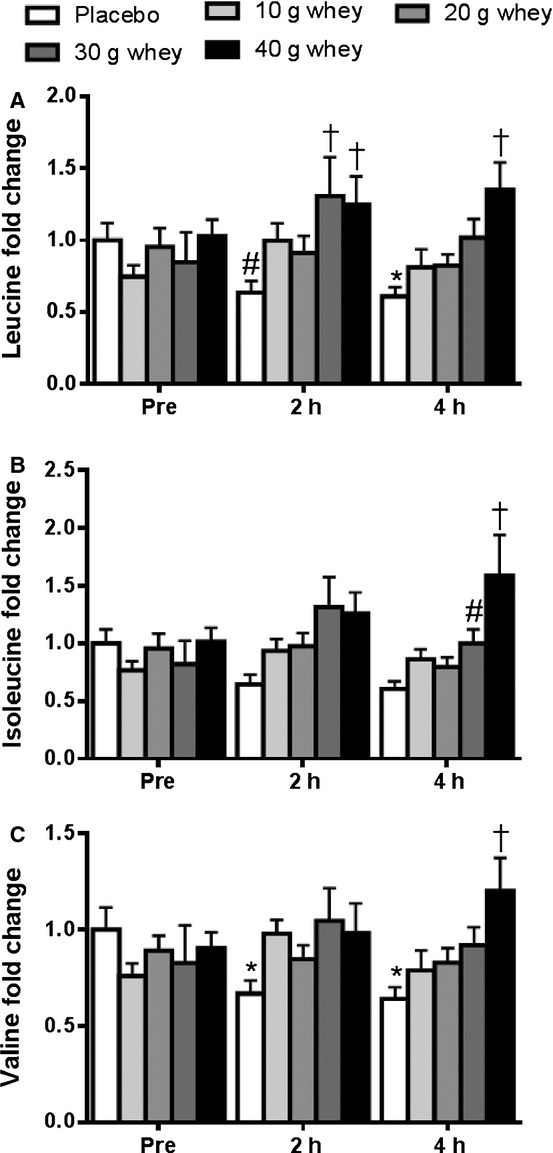
Intramuscular branch chain amino acids. Fold changes in (A) leucine, (B) isoleucine, and (C) valine at rest, 2 h, and 4 h post resistance exercise. *significantly different from pre‐exercise within the same treatment *P* < 0.05. ^#^trend toward difference from pre‐exercise within the same treatment. ^†^significantly different from placebo condition within the same time point.

Figure 1 displays a heat map of the changes in intramuscular amino acids in response to resistance exercise and whey protein ingestion which we were able to resolve by GC‐MS. Significant changes in the EAAs were limited to histidine and the three BCAAs; leucine, isoleucine and valine. Intramuscular leucine decreased below fasting levels at 4 h post exercise in the placebo group (*P* < 0.05), with a strong trend toward a decrease at the 2 h time point (*P* = 0.06) as well (Fig. 2A). The significant drop in muscle leucine during postexercise recovery was not observed in the protein supplemented groups, and leucine levels were elevated intramuscularly in the 30 g and 40 g protein supplemented groups when compared to the placebo group at 2 h post exercise (*P* < 0.05), as well as for the 40 g group only at 4 h post exercise (*P* < 0.001). Similarly, intramuscular isoleucine levels (Fig. 2B) were elevated in the 40 g whey group compared to the placebo group at 4 h post exercise (*P* < 0.001), with similar trend observed for the 30 g whey condition (*P* = 0.06). In the placebo group, there was a drop in intramuscular valine below fasting levels at both 2 and 4 h post exercise (Fig. 2C). The decline in intramuscular valine during postexercise recovery was reversed by graded protein intake and achieved levels greater than the placebo group at the 4 h post exercise time point for those receiving in the 40 g whey supplementation group (*P* < 0.001).

**Figure 3.**
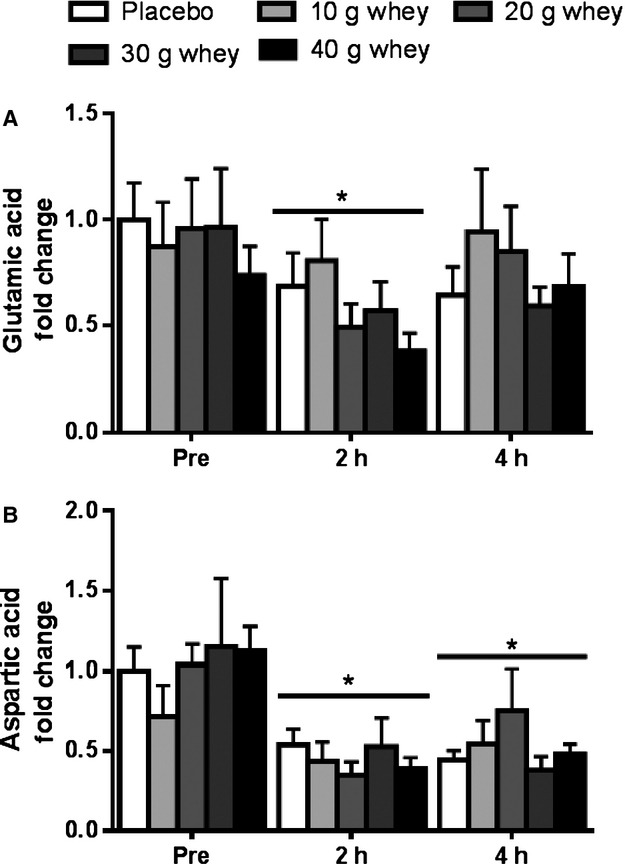
Intramuscular glutamic and aspartic acids. Fold changes in (A) glutamic acid and (B) aspartic acid. *main effect for time, significantly different from the pre‐exercise fasted condition *P* < 0.05.

Small but significant changes were observed for the NEAAs ornithine, alanine, and proline. There was significant main effect (*P* = 0.024) for greater ornithine concentrations at 4 h after exercise compared with 2 h. Elevated proline intramuscular levels were observed within the 40 g whey group at 4 h compared to 2 h post exercise (*P* = 0.002). Similarly, proline showed significantly greater levels in the 40 g whey group compared to the placebo at the 4 h post exercise time point (*P* = 0.006). At 4 h post exercise, intramuscular alanine levels were greater in the 20 g whey group compared with the placebo (*P* = 0.003) or the 10 g whey (*P* = 0.014) groups. At 4 h post exercise, intramuscular alanine levels were greater in the 20 g whey group compared with the placebo (*P* = 0.003) or the 10 g whey (*P* = 0.014) groups There was also a decrease in intramuscular glutamic acid at 2 h (*P* < 0.001) post exercise regardless of the supplement condition (Fig. 3A). Similarly, resistance exercise alone induced a decrease in intramuscular aspartic acid at both 2 and 4 h post exercise (*P* < 0.001) in all the supplement conditions (Fig. 3B). 4‐Methyl‐2‐oxopentanoic acid (or *α*‐ketoisocaproic acid, *α*‐KIC) was unchanged with the exception of lower intramuscular levels at 2 h post exercise in the 10 g whey group only (data not shown). The heat map (Fig. 1) also shows that the mean concentrations of some AAs did also tend to increase in whey supplemented groups during postexercise recovery as indicated by a red color, however, if not noted above these changes did not achieve statistical significance (*P* > 0.05).

Strong correlations were observed among intramuscular levels of the BCAAs in response to resistance exercise and whey protein supplementation. At the 2 h postexercise time point, significant linear correlations were observed between intramuscular leucine and isoleucine (*r* = 0.98, *P* < 0.0001) leucine and valine (*r* = 0.95, *P* < 0.0001), and between isoleucine and valine (*r* = 0.93, *P* < 0.0001). These correlations indicate that the BCAAs display similar kinetics and are metabolized in a similar manner.

### p70S6K Thr389 phosphorylation

**Figure 4.**
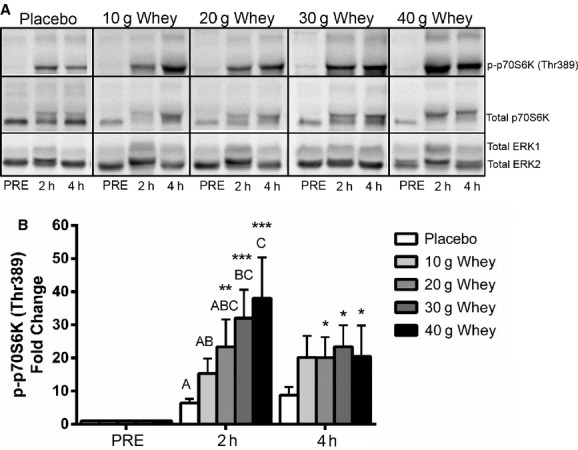
p70S6 kinase phosphorylation. Panel (A) shows representative western blots for p‐ p70S6K (Thr38), total p70S6K, and total ERK1 and 2 because of the large mobility shift in total p70S6K associated with phosphorylation total ERK2 was used as a loading control. Panel (B) shows fold change in p70S6K phosphorylation from the pre‐exercise fasted condition. *Significantly different from pre in the same condition *P* < 0.05. **Significantly different from pre in the same condition *P* < 0.01. ***Significantly different from pre in the same condition *P* < 0.001 Within the 2 h post exercise condition treatments are significantly different from those with a different letter *P* < 0.05.

Figure 4 displays the fold change in p70S6K Thr389 phosphorylation during postexercise recovery in response to graded intakes of whey protein. There was a significant time × group interaction; post hoc testing revealed that p70S6K phosphorylation was significantly elevated above fasting levels at 2 h post exercise only for groups receiving ≥20 g of whey protein supplementation. The fold change from baseline in p70S6K Thr389 phosphorylation was additionally significantly greater at 2 h post exercise in response to 40 g whey intake when compared to either the placebo or 10 g whey groups (*P* < 0.05). Furthermore, ingestion of 30 g of whey protein was found to result in significantly greater p70S6K Thr389 phosphorylation when compared to the placebo condition only (*P* < 0.05). Overall, linear regression analysis revealed a significant dose‐dependent relationship at 2 h post exercise between whey protein dose ingested and the extent of p70S6K Thr389 phosphorylation (*r* = 0.51, *P* < 001).

**Figure 5.**
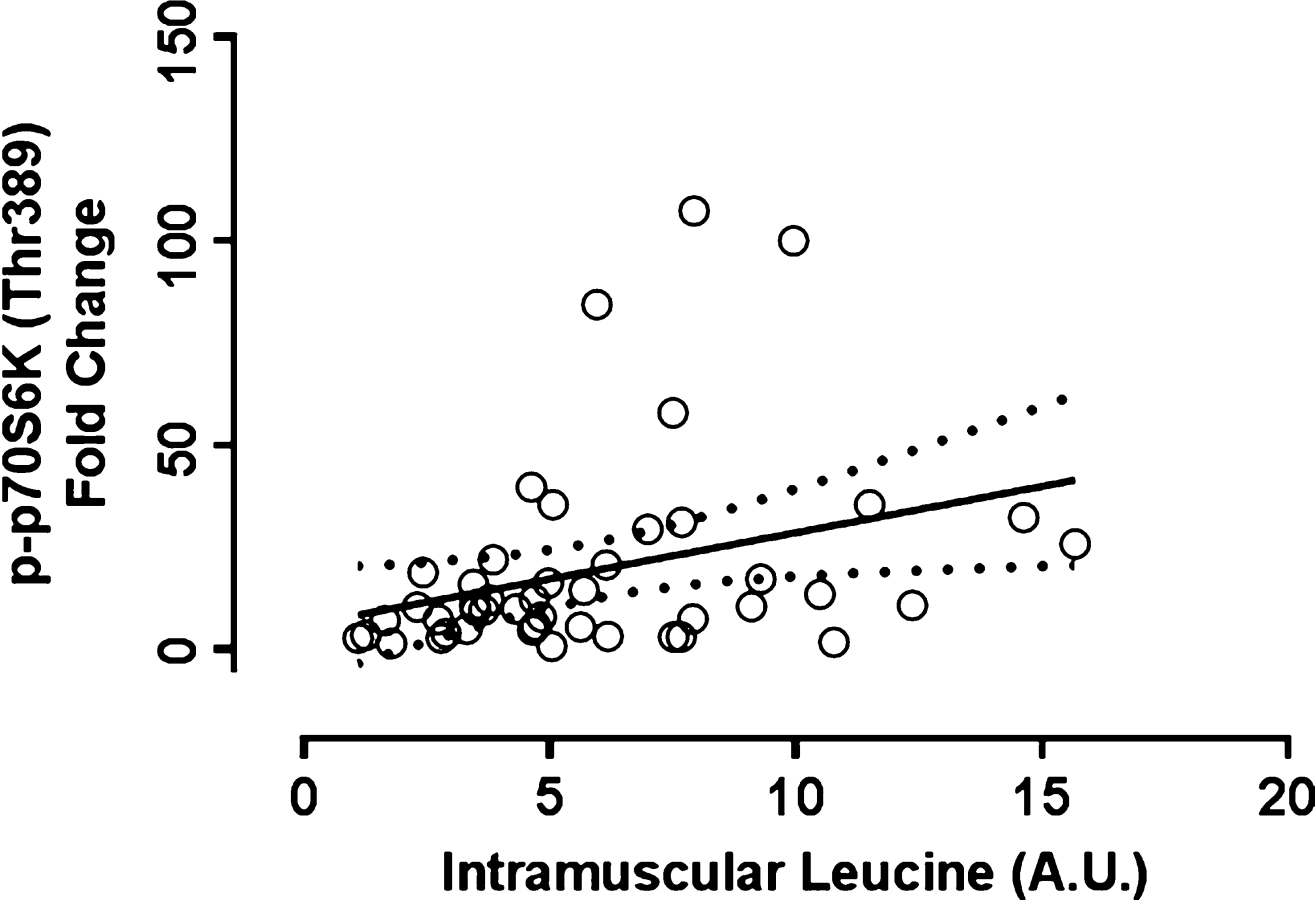
Relationship between p70S6 Kinase Phosphorylation and Intramuscular Leucine. Fold change from rest of p70S6K phosphorylation at 2 h post exercise was significantly correlated with intramuscular leucine content at 2 h post exercise *r* = 0.32, *P* = 0.026. The solid line represents the line of best fit as determined by linear regression and the dotted lines represent 95% confidence intervals.

Intramuscular levels of leucine at 2 h post exercise were found to be significantly and positively correlated with the extent of p70S6K Thr389 phosphorylation (*r* = 0.32, *P* = 0.026; Fig. 5) at 2 h of recovery. Similarly, this relationship was found to be significant for isoleucine (*P* = 0.017, *r* = 0.33, data not shown), and exhibit a trend toward significance for valine (*P* = 0.063, *r* = 0.27 data not shown).

## Discussion

This study shows that the dose–response relationship between whey protein intake and p70S6K phosphorylation in the muscle of older men during exercise recovery follows a similar pattern to that of the previously reported between whey protein intake and MPS (Yang et al. 2012b). Furthermore, the present study is the first to characterize the changes that occur in intramuscular BCAA concentration following resistance exercise with or without graded intakes of whey protein. The results show that an acute bout of resistance exercise results in a decrease in intramuscular BCAA concentration in older men during postexercise recovery and that the whey protein supplementation can reverse this decline.

The reduction in intramuscular BCAA content after resistance exercise observed in the present study may be explained by three different, but not mutually exclusive, fates: (1) utilization for protein synthesis; (2) conversion to their respective branched‐chain *α*‐keto acids and subsequent release into the bloodstream; or (3) oxidization for energy. (Shimomura et al. 2004; Brosnan and Brosnan 2006). In a murine model, high‐resistance muscular contractions performed in the fasted state have been shown to promote an influx of BCAAs, especially leucine, into the muscle (MacKenzie et al. 2009). Conversely, we found that the resistance exercise performed by older men in the fasted state in the absence of postexercise protein provision results in a decrease in intramuscular BCAA levels. The lack of change observed in intramuscular 4‐Methyl‐2‐oxopentanoic acid (or *α*‐ketoisocaproic acid, *α*‐KIC) might indicate that BCAAs were being used for protein synthesis. However, it is also possible that BCAA metabolites were released from the muscle or were oxidized locally at a similar rate to which they were produced. Indeed, at the whole body level, protein supplementation has been shown to increase leucine oxidation during postexercise recovery (Yang et al. 2012b). Because we did not measure *α*‐KIC in other tissue pools or leucine oxidation in the current study, we cannot make definitive claims about the final metabolic fate of the elevated intramuscular BCAAs.

There has been a considerable interest into the optimal dose of protein ingestion required to maximize muscle anabolism during postexercise recovery (Moore et al. 2009; Yang et al. 2012b; Witard et al. 2014). We found that as little as 10 g of whey protein was able to prevent the significant decline in intramuscular BCAA levels during postexercise recovery. However, only the larger doses of whey (30 and 40 g) were able to increase BCAA levels significantly above that seen in the placebo condition. Wijekoon et al. (Wijekoon et al. 2004) have shown a strong correlation among the concentrations of all three BCAAs in plasma of rats. We extend this finding by showing a similarly strong correlation between the levels of the individual BCAAs in human muscle tissue, suggesting that they share similar kinetics.

We found that whey protein supplementation also increased the phosphorylation of p70S6k at Thr389 in a dose‐dependent manner. Although not statistically significant, there was ~15% greater p70S6K phosphorylation after the ingestion of 40 g of whey compared to 30 g suggesting that a plateau may not have been reached. Additionally, the dose‐dependent relationship beyond a 40 g dose was supported by the overall significant linear relationship between p70S6K Thr389 phosphorylation and whey dose ingested. The linear relationship between whey protein dose and muscle p70S6K phosphorylation is in agreement with the previously reported MPS data showing an increasing anabolic response up to 40 g of whey protein after resistance exercise in older men (Yang et al. 2012b). These data are in contrast to postexercise whey supplementation data in young men showing an apparent plateau in MPS response after the ingestion of whey protein dose in surplus of 20 g (Witard et al. 2014).

Among the BCAAs, leucine is thought to be the primary trigger of mTORC1 signaling (Drummond et al. 2009). Indeed, phosphorylation of p70S6k at Thr389 was found to significantly correlate with intramuscular leucine content in the present study. This correlation is in line with mechanistic data showing that intracellular leucine sensors are obligatory for mTOR pathway activation (Zoncu et al. 2011; Han et al. 2012). Muscle protein synthesis has previously been correlated to plasma leucine concentration following protein ingestion in previous studies (Pennings et al. 2011). To our knowledge, this is the first study to report a correlation of p70S6K with intramuscular leucine levels in humans. Interesting however, p70S6K phosphorylation tended to be increased during postexercise recovery in the placebo group, despite a significant reduction in muscle leucine content, suggesting that resistance exercise alone in the fasted state is capable of stimulating this pathway through a leucine independent mechanism (West and Baar 2013). Nevertheless, the replenishment of intramuscular leucine during postexercise recovery appeared to promote an even greater activation of mTORC1 pathway as evidenced by heightened p70S6K phosphorylation. While p70S6K phosphorylation has been found to correlate with changes to MPS in young adults, evidence suggests some disconnect in the muscle of older subjects (Kumar et al. 2009; Fry et al. 2011). Therefore, anabolic resistance within senescent muscle may be related both to the ability of leucine to activate p70S6K (Cuthbertson et al. 2005) as well as disconnect between mTOR pathway activation and the resulting MPS response. The impairment of MPS in the muscle of elderly subjects in response to feeding and contraction has been controversial, with different studies reporting opposing findings (Fry et al. 2011; Burd et al. 2013; Kiskini et al. 2013). This heterogeneity in study findings may be due to a number of lifestyle factors such as long‐term diet and physical activity patterns which may promote dissociation between chronological age and anabolic sensitivity (Churchward‐Venne et al. 2013a).

While the plasma amino acid response to protein feeding, with or without resistance exercise, has been routinely measured, intramuscular amino acid responses have received much less attention. Bohé et al. (Bohe et al. 2003) concluded that at rest the infusion of amino acids led to a large rise in plasma EAA content, but a decrease in the levels of EAA locally within the skeletal muscle tissue. These results were extrapolated to hypothesize that extracellular, rather than intracellular EAA content, are the primary regulators of the anabolic response to protein feeding. Nevertheless, Bohé et al. measured only aggregate EAA responses and did not report changes in individual AAs or BCAAs (Bohe et al. 2003). This study also shows greater intracellular EAA concentration compared with plasma EAA levels suggesting that EAAs must travel up a concentration gradient to enter the muscle cell. More recent studies have replicated the finding of greater EAA concentration intramuscularly compared to in circulation but have found that BCAAs, and in particular leucine, are found at higher concentrations in the plasma when compared with the muscle sarcoplasm (Churchward‐Venne et al. 2012, 2014). Taken together, these results suggest that BCAAs are regulated in a different manner than the other EAAs. On this basis, it is important to consider the concentration of the BCAA independent from other EAAs.

Previous studies have shown that in young and older men, (Drummond et al. 2011) resistance exercise in the fasted state reduces intramuscular leucine content. Furthermore, in young men the provision of protein containing leucine after resistance exercise has been reported to result in the maintenance of intramuscular leucine content during postexercise recovery (Churchward‐Venne et al. 2012, 2014). The present study expands on these findings by showing that in older men the provision of whey protein reverses the drop in intramuscular leucine and other BCAAs caused by resistance exercise. Intramuscular leucine did not increase in a clear linear fashion with increasing doses of whey protein, but did appear to explain ~10% of the variance in p70S6K phosphorylation. There are two likely explanations for the relatively low proportion of the variance explained in p70S6K phosphorylation by intramuscular leucine levels. Firstly, recent work suggests that the lumen of the lysosomes may be the cellular location where amino acids (primarily leucine) are sensed, so the direct measurement of this subcellular concentration might yield a stronger correlation with anabolic response (Zoncu et al. 2011). Secondly, while aging results in an anabolic resistance to feeding, it is likely that related decreases in physical activity and increased inflammation mediate this anabolic resistance rather than the chronological age per se (Churchward‐Venne et al. 2013b). This assertion is partially supported by the relationship between intramuscular leucine and p70S6K phosphorylation (Fig. 5) observed in the present study, which appeared to show that some subjects (those falling above the trend line) appear to possess a higher anabolic sensitivity and are able to mount a larger p70S6K phosphorylation response to smaller changes in intramuscular leucine levels. On the other hand, there was a subset of subjects (below the trend line) who exhibited more modest augmentation of the p70S6K response in the presence of a high intramuscular leucine levels.

Only very minor changes were observed in most of the nonessential amino acids measured in human muscle in the present study. However, both aspartic acid and glutamic acid were found to be reduced intramuscularly following resistance exercise, even following the ingestion of the maximally tested 40 g dose of whey protein. This is a somewhat surprising result given that glutamic acid and aspartic acid are two of the most abundant amino acids in whey protein. The unresponsiveness of intramuscular aspartic and glutamic acid to protein supplementation may be explained by the fact that most of dietary aspartic acid and glutamic acid are retained and utilized by the gastrointestinal tissues such that they may not be available to the muscle in large amounts following feeding (Stoll and Burrin 2006). Nevertheless, despite low intramuscular aspartic acid and glutamic acid availability, subjects were still able to mount a robust anabolic response as assessed by p70S6K activation suggesting a disconnect between these particular NEAAs and muscle anabolic signaling during postexercise recovery.

## Conclusion

This study shows that in older men resistance exercise in the fasted state results in a decrease in intramuscular BCAA content, but does not appear to influence the local levels of the remaining EAAs. As little as 10 g of whey protein supplementation was able to prevent the decrease in intramuscular BCAAs that occurred when resistance exercise was performed in the fasted state. While our results suggest that larger doses 30–40 g of whey protein were required to significantly increase intramuscular BCAA levels above those seen with only placebo ingestion, p70S6K phosphorylation was increased in a dose‐dependent manner by ingestion of up to 40 g of whey protein. This result is in agreement with recently reported MPS data which show that in contrast to young men, older subjects may require more than the 20 g of high‐quality protein in order to maximize the muscle anabolic response during the postexercise recovery.

## Acknowledgments

The authors would like to thank the study volunteers. The authors are also exceptionally grateful for the medical support supplied by A. Garnham, who undertook all muscle biopsy procedures and M. Frankish who assisted with the clinical study.

## Conflict of Interest

None declared.
